# Estradiol signaling mediates gender difference in visceral adiposity via autophagy

**DOI:** 10.1038/s41419-018-0372-9

**Published:** 2018-02-22

**Authors:** Zhipeng Tao, Louise D. Zheng, Cayleen Smith, Jing Luo, Alex Robinson, Fabio A. Almeida, Zongwei Wang, Aria F. Olumi, Dongmin Liu, Zhiyong Cheng

**Affiliations:** 1 Department of Human Nutrition, Foods, and Exercise, Fralin Life Science Institute, College of Agriculture and Life Science, Virginia Tech, Blacksburg, VA 24061 USA; 2 Department of Health Promotion, Social & Behavioral Health, College of Public Health, University of Nebraska Medical Center, Omaha, NE USA; 3 Department of Urology, Massachusetts General Hospital, Harvard Medical School, Boston, MA USA

## Abstract

Excessive adiposity (particularly visceral fat mass) increases the risks of developing metabolic syndrome. Women have lower deposit of visceral fat than men, and this pattern becomes diminished postmenopausally, but the underlying mechanism remains largely unknown. Here, we show that the gender difference in visceral fat distribution is controlled by an estradiol–autophagy axis. In C57BL/6J and wild-type control mice, a higher visceral fat mass was detected in the males than in the females, which was associated with lower expression of estrogen receptor α (ERα) and more active autophagy in males vs. females. However, deletion of ERα normalized autophagy activity and abolished the gender difference in visceral adiposity. In line with the adiposity-reducing effect of the ERα–autophagy axis, we found that downregulation of ERα and increased autophagy activity were required for adipogenesis, while induction of estradiol signaling dampened autophagy and drastically prevented adipogenesis. Mechanistically, the estradiol-ERα signaling activated mTOR, which phosphorylated and inhibited ULK1, thereby suppressing autophagy and adipogenesis. Together, our study suggests that the lower visceral adiposity in the females (vs. the males) arises from a more active estradiol-ERα signaling, which tunes down autophagy and adipogenesis.

## Introduction

White adipose (or fat) tissues (WAT) play a central role in metabolic homeostasis through energy storage and endocrine functions^1,2^. It has been shown that fat depots at distinct anatomical (e.g., subcutaneous vs. visceral) locations have intrinsic differences in hormone response, gene expression, remodeling, and metabolism^3–9^. Excessive visceral fat is associated with metabolic syndrome development (e.g., insulin resistance) in animal models and humans, whereas subcutaneous fat is benign or protective^9–13^. Compared with women, men have more visceral fat^14,15^. Intriguingly, visceral fat mass increases in post-menopausal women (characteristic of reduced estrogen secretion), which can be prevented by estrogen replacement therapy^16,17^. These findings underscored an important role of estrogen signaling in the regulation of fat development and distribution, yet the molecular mechanism remains largely elusive.

WAT mass development and maintenance are dependent on adipocyte turnover. It was estimated that the rate of adipocyte turnover was 10% per year in humans and 1–5% per day in mice^18,19^. The overall adipocyte number in WAT is balanced by adipogenesis (i.e., the differentiation of preadipocyte into adipocytes) and adipocyte apoptosis^18–20^. Emerging evidence suggests that autophagy, the major intracellular degradation and remodeling system, regulates both adipocyte differentiation and apoptosis^21–23^. Blockage of autophagy through ablation of Atg5 or Atg7 substantially prevents adipocyte differentiation and promotes adipocyte apoptosis, which significantly reduces fat mass in mice^21–23^. We and others have showed that autophagy is required to maintain PPARγ and FSP27, the key regulators of adipocyte differentiation and lipid droplet formation in fat cells^24,25^. Suppression of autophagy downregulates PPARγ and FSP27, concomitant with dampened adipocyte differentiation and marginal lipid accumulation in the cells^24,25^. Therefore, autophagy acts as a critical regulator of WAT remodeling and maintenance.

Given the above-mentioned evidence that implies estrogen and autophagy in WAT regulation, we asked the question whether autophagy might interact with estrogen signaling, and how it might link to the gender difference in visceral adiposity. Here, we show that male mice had higher visceral fat mass than the females, which was associated with lower expression of estrogen receptors (ERα) in the visceral adipose tissue. Activation of estradiol (E2) signaling suppressed autophagy via an mTOR-ULK pathway, which inhibited adipogenesis and was associated with a lower visceral adiposity in the mice. However, deletion of ERα normalized autophagy activity and gender-dependent difference in visceral adiposity. Our data reveals for the first time an E2–ERα–autophagy axis that contributes to the gender difference in visceral fat distribution.

## Results

### Female mice had lower visceral WAT (vWAT) mass than male mice

**Fig. 1:**
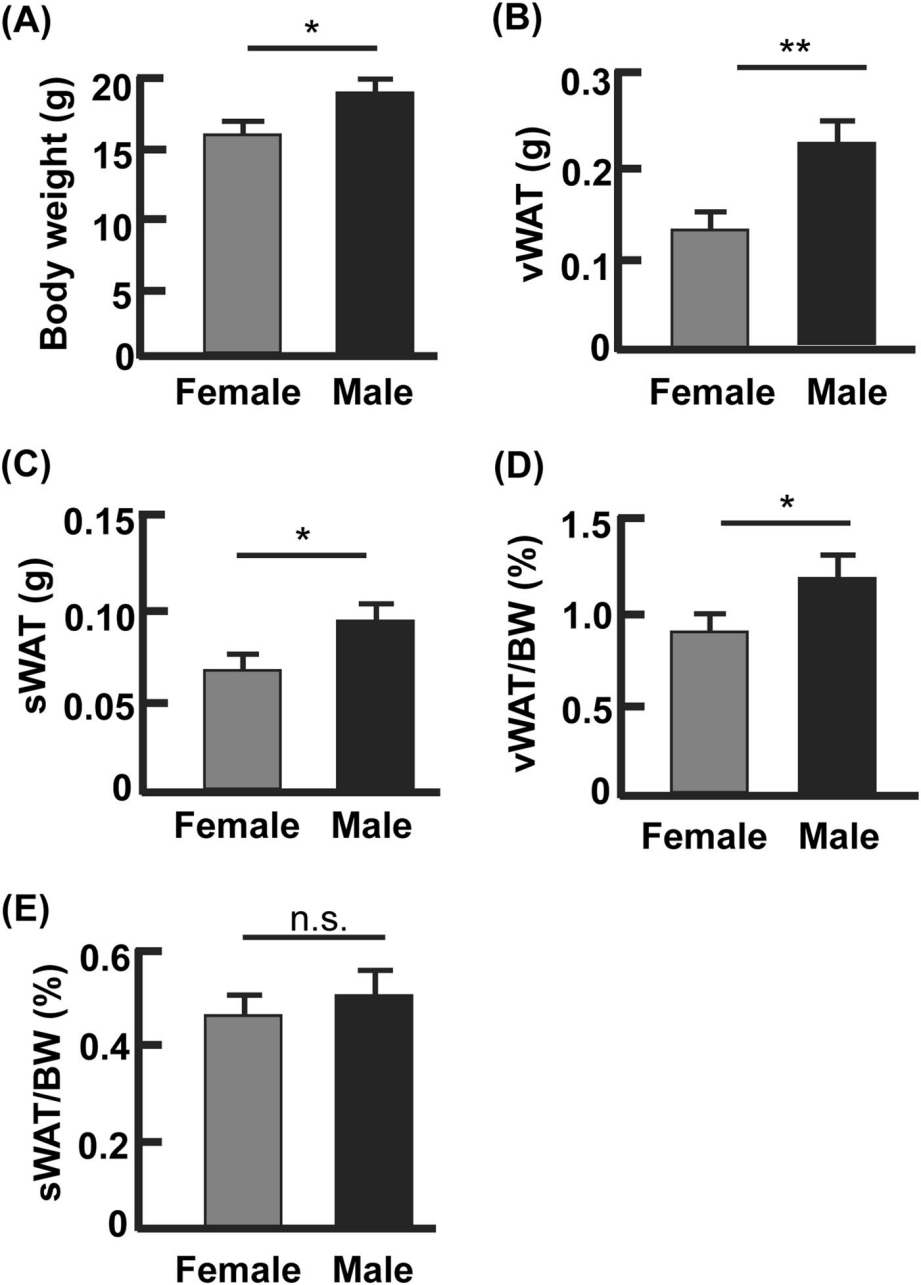
Gender difference existed in the percentage of visceral but not subcutaneous fat. **a** The body weights of male and female mice at the age of 6–7 weeks. **b** The net weights of visceral WAT from male and female mice. **c** The net weights of subcutaneous WAT from male and female mice. **d** The percentage of visceral WAT from male and female mice, normalized against the body weights. **e** The percentage of subcutaneous WAT from male and female mice, normalized by body weights. *p < 0.05; **p < 0.01; n.s. not significant; *n* = 4–6

A higher vWAT volume in men than women has been observed across races^14,15^. To determine if mice have a similar pattern of fat distribution, we examined C57BL/6J at the age of 6–7 weeks (Fig. 1). As expected, the female mice had lower body weight than males (average 15.4 g vs. 17.5 g, *p* < 0.05; Fig. 1a). The net weights of gonadal WAT (the largest visceral fat depots in mice) and subcutaneous WAT (inguinal fat depots, sWAT) were both lower in female mice than in the males (Fig. 1b, c). However, after normalization against the body weight only the vWAT mass remained lower in females than in males (average 0.87 vs. 1.21%, *p* < 0.05; Fig. 1d), while the differences in sWAT mass became indiscernible between the males and females (average 0.45 vs. 0.49%, *p* = 0.14; Fig. 1e). Therefore, the ratios of visceral fat to body weight (or vWAT percentage) reveal a gender-dependent phenotype in mice as observed previously in human subjects.

### Autophagy activity in vWAT was lower in female than male mice

**Fig. 2:**
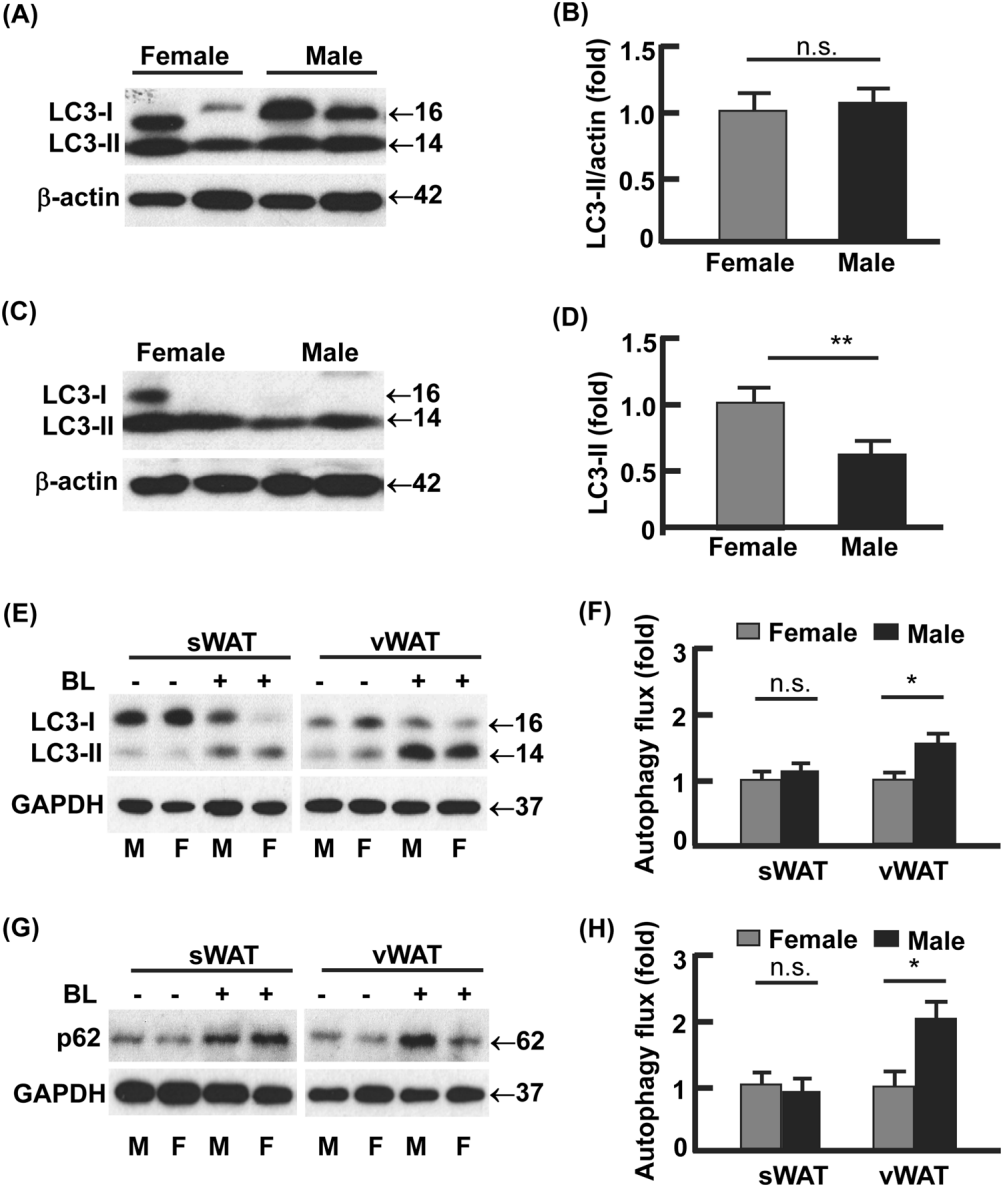
Autophagy activities showed gender difference in visceral but not subcutaneous fat. **a**, **b** The steady-state protein levels of LC3 in subcutaneous WAT, analyzed by Western blotting (**a**) and densitometry (**b**). **c**,** d** The steady-state protein levels of LC3 in visceral WAT, analyzed by Western blotting (**c**) and densitometry (**d**). **e**–**h** Measurement of autophagy flux in subcutaneous and visceral WAT. The WAT explant cultures were incubated with and without autophagy inhibitor bafilomycin A1 (0.1 μM) and leupeptin (10 μg/ml) for 4 h, and the turnovers of LC3-II and p62 were examined by Western blotting (**e**, **g**) and densitometry (**f**, **h**). In densitometric analyses, the band densities of investigated proteins were normalized against that of GAPDH or β-actin, and the fold changes were calculated by taking the normalized density of female group as “1”. For autophagy flux, we first normalized the band densities of LC3-II and p62 against that of GAPDH, then calculated the differences of normalized densities in the presence vs. the absence of autophagy inhibitor; lastly, the differences were shown as fold changes by taking the female group as “1”. BL bafilomycin A1 and leupeptin, M male, F female; *p < 0.05; **p < 0.01; n.s. not significant; *n* = 3–4

Autophagosome formation is characterized by lipidation of LC3 to form LC3-phospholipid conjugate (LC3-II), which can be degraded by lysosomal hydrolase in autolysosome^26,27^. In sWAT, steady-state LC3-II levels did not differ between female and male mice (Fig. 2a, b). However, a significantly lower (50%, *p* < 0.01) steady-state LC3-II was detected in vWAT from male mice vs. that from female mice (Fig. 2c, d), suggesting that autophagic degradation of LC3-II in vWAT was more active in the males than in the females. To test this, we detected LC3-II turnover (or autophagy flux)^25,27,28^, by detecting the accumulation of LC3-II after treating WAT explant cultures with autophagy inhibitors bafilomycin A1 and leupeptin (BL) for 4 h. In line with the steady-state levels of LC3-II in sWAT being indiscernible between the males and females (Fig. 2a, b), autophagy flux in sWAT did not show significant difference between the males and females (Fig. 2e, f). However, the autophagy flux in vWAT was significantly higher in the males (1.6-fold upregulated, *p* < 0.05) than in the females (Fig. 2e, f). The turnover of p62, which is selectively degraded by autophagy, further validated the higher autophagy activity in vWAT from male mice than that from female mice (Fig. 2g, h). These data support the notion that increased LC3-II turnover results in a reduced steady-state level of LC3-II (Fig. 2c, d)^26,29^. Together, female mice had lower autophagy activity in vWAT than the males.

### Estrogen receptors were upregulated in vWAT from females vs. males

**Fig. 3:**
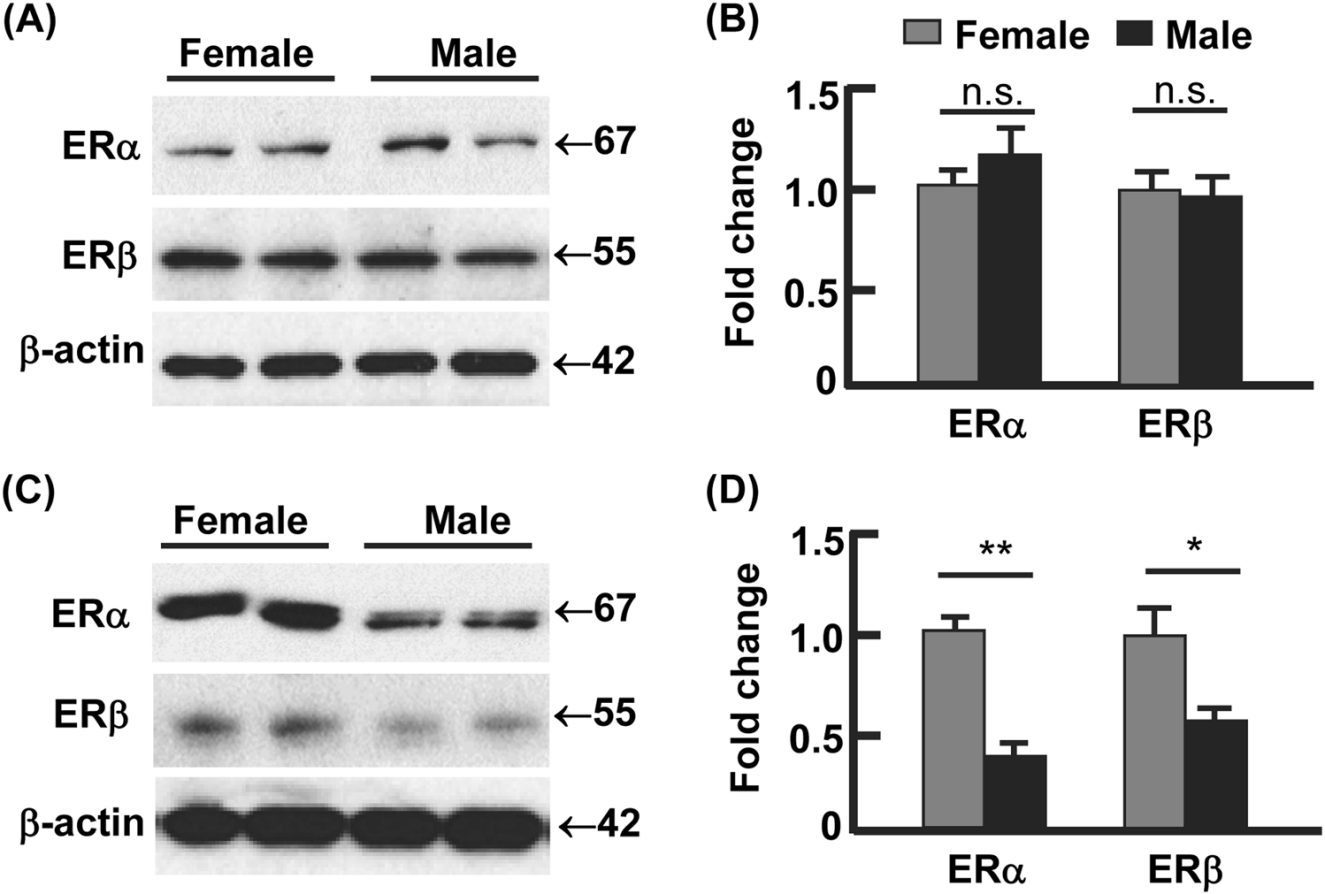
The expression of estrogen receptors (ER) showed gender difference in visceral but not subcutaneous fat. **a**–**b** The protein levels of ERα and ERβ in subcutaneous WAT, analyzed by Western blotting (**a**) and densitometry (**b**). **c**–**d** The protein levels of ERα and ERβ in visceral WAT, analyzed by Western blotting (**c**) and densitometry (**d**). In densitometric analysis, the band densities of investigated proteins were normalized against that of β-actin, and the fold changes were calculated by taking the normalized density of female group as “1”. *p < 0.05; **p < 0.01; n.s. not significant; *n* = 3–4

E2 signaling is primarily funneled through ERα and ERβ^30^. Compared with the males, the female mice had similar expression of ERα and ERβ in sWAT (Fig. 3a, b). However, vWAT had significant lower protein levels of ERα and ERβ in male mice than in female mice (Fig. 3c, d), being 40% for ERα (*p* < 0.01) and 54% for ERβ (*p* < 0.05). Interestingly, the overall protein levels of ERβ in vWAT appeared to be much lower than that of ERα for both genders (Fig. 3c, d), suggesting that ERα might play a dominant role in mediating E2 actions in vWAT.

### Adipogenesis was associated with downregulation of estrogen receptors but upregulation of autophagy activity

**Fig. 4:**
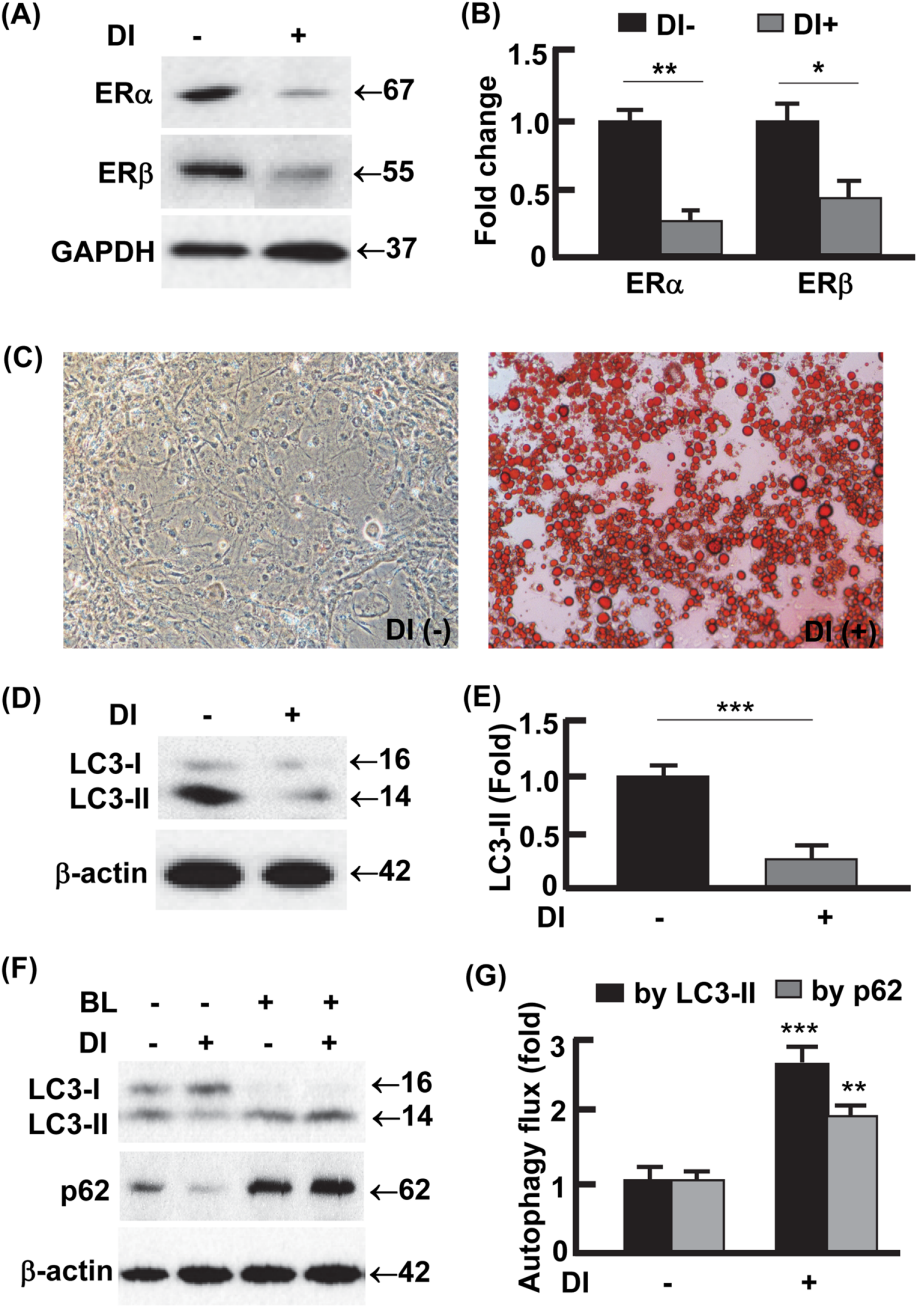
Adipogenesis was associated with downregulation of ER but upregulation of autophagy activity. **a**–**b** The protein levels of ERα and ERβ in preadipocytes and mature (or differentiated) adipocytes, analyzed by Western blotting (**a**) and densitometry (**b**). DI differentiation induction. DI+ represents differentiated 3T3L1 cells (mature adipocytes) harvested on day 12; DI− represents 3T3L1 cells without differentiation induction (i.e., preadipocytes) harvested on day 12. **c** Oil Red O staining to detect the differentiation of preadipocytes into mature adipocytes. On day 12, massive lipid accumulation was detected in mature adipocytes but not in preadipocytes. **d**, **e** The steady-state protein levels of LC3 in preadipocytes and mature adipocytes, analyzed by Western blotting (**d**) and densitometry (**e**) on day 12. **f**, **g** Measurement of autophagy flux in preadipocytes and mature adipocytes. On day 12, the cells were incubated in the presence or absence of autophagy inhibitor BL (bafilomycin A1 at 0.1 μM and leupeptin at 10 μg/ml) for 4 h, and the turnovers of LC3-II and p62 were examined by Western blotting (**f**) and densitometry (**g**). In densitometric analysis, the band densities of investigated proteins were normalized against that of GAPDH or β-actin, and the fold changes were calculated by taking the normalized density of DI− group as “1”. For autophagy flux analysis, we first normalized the band densities of LC3-II and p62 against that of β-actin, then calculated the differences of normalized densities in the presence vs. the absence of autophagy inhibitor; lastly, the differences were shown as fold changes by taking the DI− group as “1”. BL bafilomycin A1 and leupeptin. *p < 0.05; **p < 0.01; ***p < 0.0001; *n* = 3–4

The observation of lower expression of ER but higher autophagy activity in the males vs. females (Figs. 2, 3) prompted us to examine whether this reciprocal relation exists in adipogenesis, the process that is critical for adipose tissue development and maintenance^31,32^. As shown in Fig. 4a and b, ERα was downregulated by 80% (*p* < 0.01) and ERβ by 54% (*p* < 0.05) during adipogenesis, which was characterized by drastic accumulation of lipid in the cells (Fig. 4c). In addition, the downregulation of ERs was associated with 81% (*p* < 0.0001) reduction in the steady-state level of LC3-II (Fig. 4d, e). Autophagy flux assays by LC3-II turnover suggested that the differentiated adipocytes had an autophagy activity 2.6-fold (*p* < 0.0001) higher than the preadipocytes, which was further verified by the turnover of p62 (Fig. 4f, g). These results recapitulated the pattern observed in vWAT, where steady-state level of LC3-II was reduced due to increased autophagy activity (Fig. 2)^26,29^. Importantly, the *in vitro* and *in vivo* data work in concert to reveal a reciprocal relationship between autophagy activity and ER levels.

### Estradiol signaling suppressed autophagy and adipogenesis

**Fig. 5:**
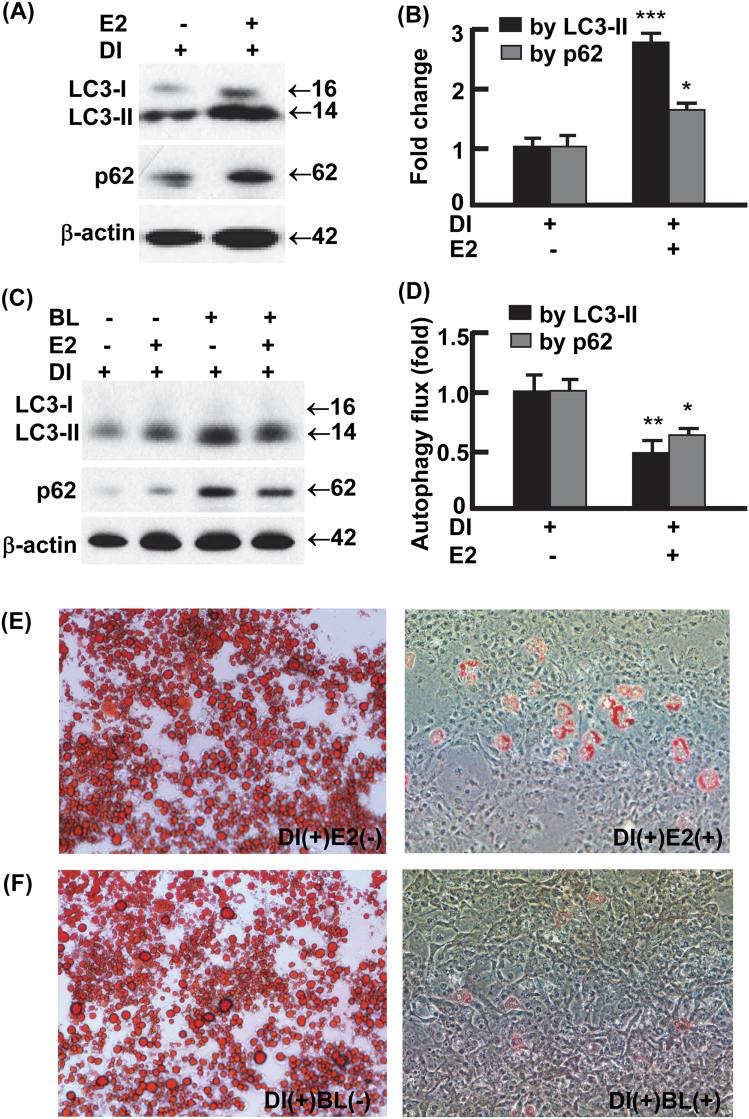
Activation of estrogen signaling suppressed autophagy and adipogenesis. **a**, **b** Estradiol (0.1 μM, days 0–12) increased the steady-state protein levels of LC3 and p62 during 3T3L1 preadipocyte differentiation, analyzed by Western blotting (**a**) and densitometry (**b**). DI+ represents 3T3L1 cells with differentiation induction and harvested on day 12. **c**, **d** The presence of E2 (0.1 μM, days 0–12) reduced autophagy activity during preadipocyte differentiation. On day 12, autophagy flux was analyzed by Western blotting (**c**) and densitometry (**d**) after the cells were incubated with and without autophagy inhibitor BL (bafilomycin A1 at 0.1 μM and leupeptin at 10 μg/ml) for 4 h. In densitometric analysis, the band densities of investigated proteins were normalized against that of β-actin, and the fold changes were calculated by taking the normalized density of E2- group as “1”. For autophagy flux analysis, we first normalized the band densities of LC3-II and p62 against that of β-actin, then calculated the differences of normalized densities in the presence vs. the absence of autophagy inhibitor; lastly, the differences were shown as fold changes by taking the E2- group as “1”. **e** The presence of E2 (0.1 μM, days 0–12) suppressed the differentiation of 3T3L1 preadipocytes, analyzed by Oil Red O staining on day 12. **f** The presence of autophagy inhibitor bafilomycin A1 (4 nM) and leupeptin (0.4 ng/ml) during days 0–12 recapitulated the effects of E2 on adipogenesis, analyzed by Oil Red O staining on day 12. *n* = 3–4; *p < 0.05; **p < 0.01; ***p < 0.0001 (with E2 vs. without E2)

To examine whether E2 signaling per se regulate adipocyte autophagy and adipogenesis, we treated 3T3L1 preadipocytes with or without E2 (0.1 μM) on day 0 through day 12, during which adipogenesis was induced according to an established protocol (see Materials and Methods)^25,33–35^. E2 treatment substantially increased the accumulation of LC3-II (2.8-folds, *p* < 0.0001), as well as p62 (1.6-fold, *p* < 0.05), the selective substrate of autophagy for degradation (Fig. 5a, b)^25,33,36^. The E2-enhanced accumulation of LC3-II and p62 was associated with reduced autophagy flux (Fig. 5c, d), suggesting that E2 dampens autophagy activity. In addition, the E2-treated cells were barely differentiated into mature adipocytes and showed marginal lipid accumulation compared with vehicle-treated cells (Fig. 5e). Likewise, treatment of 3T3L1 preadipocytes with the established autophagy inhibitor BL almost completely prevented adipogenesis (Fig. 5f). In addition, the presence of E2 or autophagy inhibitor BL similarly inhibited autophagy and adipogenesis in primary stromal vascular cells isolated from vWAT (Fig. 1s). Thus, E2 signaling suppresses adipogenesis at least in part via autophagy inhibition.

### Estradiol signaling suppressed autophagy via mTOR-ULK1

**Fig. 6:**
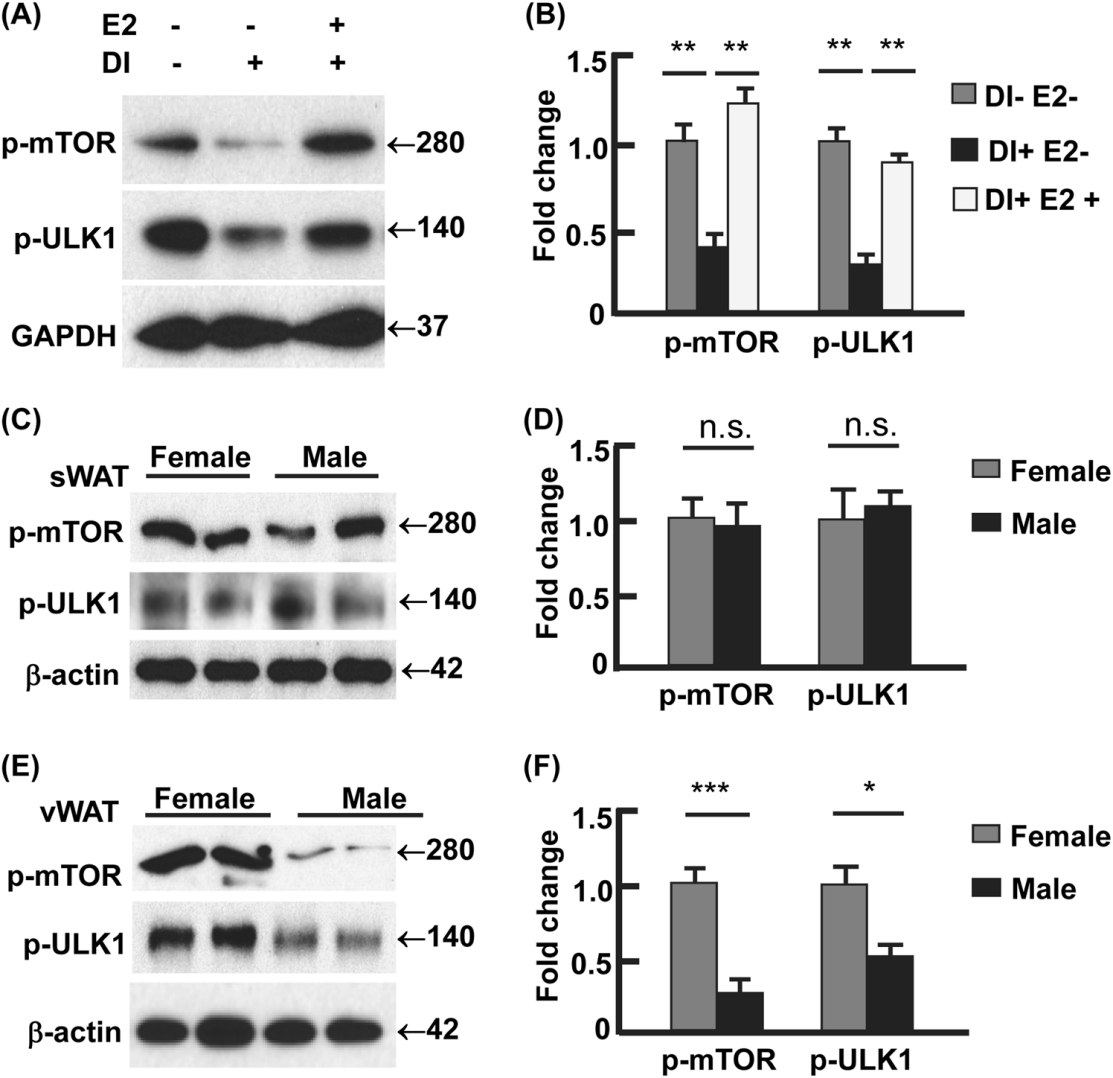
Estradiol-ER signaling regulated autophagy via mTOR-ULK1 pathway. **a**, **b** E2 (0.1 μM, days 0–12) induced the activating phosphorylation of mTOR (Ser2448) and the mTOR-catalyzed inhibitory phosphorylation of ULK1 (Ser757), analyzed by Western blotting (**a**) and densitometry (**b**). DI+ represents 3T3L1 cells with differentiation induction and harvested on day 12, and DI− represents 3T3L1 cells without differentiation and harvested on day 12. **c**, **d** The females and males showed comparable phosphorylation of mTOR (Ser2448) and ULK1 (Ser757), analyzed by Western blotting (**c**) and densitometry (**d**). **e**, **f** The females showed significantly stronger phosphorylation of mTOR (Ser2448) and ULK1 (Ser757) than the males, analyzed by Western blotting (**e**) and densitometry (**f**). In densitometric analysis, the band densities of investigated proteins were normalized against that of GAPDH or β-actin, and the fold changes were calculated by taking the normalized density of DI-E2- group (**a** and **b**) or female group (**c**–**f**) as “1”. *p < 0.05; **p < 0.01; ***p < 0.0001; n.s. not significant; *n* = 3–4

To explore the mechanism of E2 regulating autophagy, we analyzed the interactions of E2 signaling and proteins that are known to control autophagy, including ULK1, beclin 1, Atg5, Atg7, and Atg12^37–39^. We found that beclin 1 was upregulated during adipocyte differentiation, but E2 treatment had marginal effect on beclin 1 level (Fig. 2s, A–B). ULK1 was activated during adipogenesis, because the mTOR-mediated inhibitory phosphorylation of ULK1 at Ser 757 (p-ULK1^Ser757^) was significantly reduced^37–39^. However, E2 treatment suppressed ULK1 by increasing p-ULK1^Ser757^, concomitant with the activation of mTOR indicated by phosphorylation at Ser2448 (p-mTOR^Ser2448^) (Fig. 6a, b)^37–41^. These data suggest that E2 signaling acts on ULK1 but not beclin 1, although both proteins participate in autophagy initiation (i.e., formation of the isolation membrane)^37–39^. Moreover, no discernible change was detected in Atg5, Atg7, and Atg12-Atg5 conjugate, the proteins or components that regulate membrane elongation^37,39^, during adipocyte differentiation or during E2 treatment (Fig. 2s, A–B). These results, along with the above observation that E2 suppressed autophagy in adipocytes (Figs. 4–5), suggest that E2 may regulate autophagy via the mTOR-ULK1 cascade.

To validate the E2/ER signaling-mTOR-ULK1 pathway *in vivo*, we examined adipose tissues from male and female C57BL/6J mice (Fig. 6c, d). In line with the females having higher ER levels in vWAT than the males (Fig. 3), the activating phosphorylation of mTOR (p-mTOR^Ser2448^) was enhanced by 4.8-fold (*p* < 0.0001), and the mTOR-mediated inhibition of ULK1 (p-ULK1^Ser757^) was 2.1-fold stronger (*p* < 0.05; Fig. 6e, f). In sWAT, however, no statistically significant difference was detected (Fig. 6c, d), consistent with the male and the females showing comparable levels of ER in sWAT (Fig. 3). Notably, we did not detect significant difference in beclin 1, Atg5, Atg7, or Atg12-Atg5 conjugate, between the male and the females (Fig. 2s, C–D). Together, our *in vitro* and *in vivo* results support the hypothesis that E2/ER signaling regulates adipose autophagy via the mTOR-ULK1 pathway.

### Ablation of ERα normalized autophagy activity and abolished gender difference in visceral adiposity

**Fig. 7:**
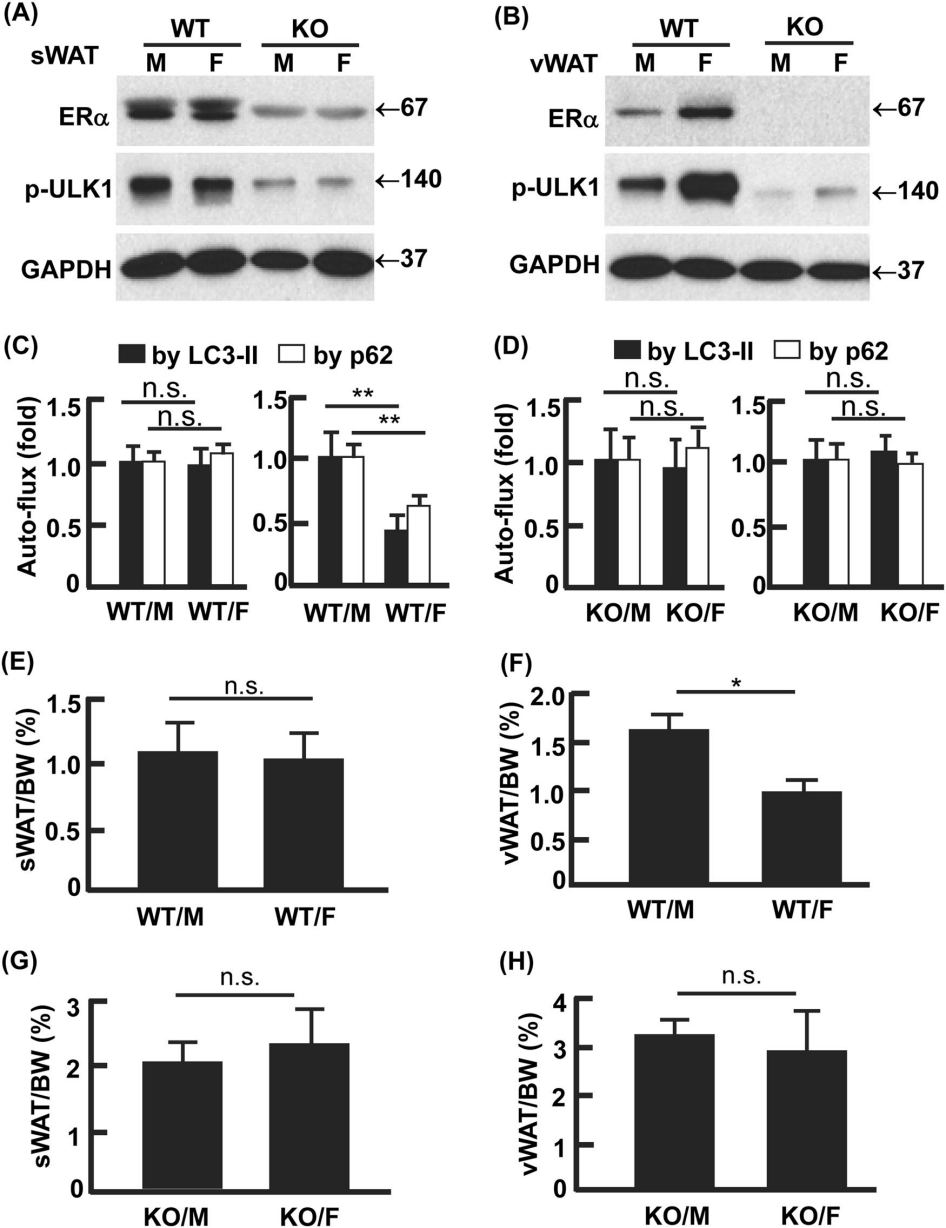
Ablation of ERα normalized gender difference in autophagy and visceral adiposity. **a** Knockout (KO) of ERα in sWAT-activated ULK1 by de-phosphorylation at Ser757. **b** KO of ERα in vWAT-activated ULK1 by de-phosphorylation at Ser757, which diminished the gender difference in ERα expression and p-ULK1 observed in the WT mice. **c**, **d** The vWAT in WT females had lower autophagy activity (LC-3II and p62 turnover) than that in WT males (**c**), but KO of ERα abolished the gender difference (**d**). The representative Western blotting images were presented in Figs. 4s and 5s. In densitometric analysis, the band densities of investigated proteins were normalized against that of GAPDH, and the fold changes were calculated by taking the normalized density of male group as “1”. For autophagy flux, we first normalized the band densities of LC3-II and p62 against that of GAPDH, then calculated the differences of normalized densities in the presence vs. the absence of autophagy inhibitor; lastly, the differences were shown as fold changes by taking the male group as “1”. **e**–**h** WT males had higher vWAT mass than the WT females (**f**), but KO of ERα abolished the gender difference (**h**). Overall, KO of ERα increased adiposity in both sWAT and vWAT, presumably due to the enhanced autophagy that promotes adipogenesis. WT wild-type, KO knockout of ERα, M male, F female, WT/M wild-type males, WT/F wild-type females, KO/M knockout males, KO/F knockout females; *p < 0.05; **p < 0.01; n.s. not significant; *n* = 5–8

ERα and ERβ have been shown to suppress or enhance autophagy in different cancer cells^42–46^. To determine the primary role player in the regulation of autophagy and adipocyte differentiation, we treated 3T3L1 cells with selective agonists of ERα (PPT) and ERβ (DPN)^47,48^. PPT reduced autophagy activity and suppressed adipogenesis but DPN had marginal effect, suggesting that ERα played the dominant role (Fig. 3s). To validate this, we examined autophagy and visceral adiposity in ERα knockout (KO) mice (Fig. 7)^49^. As observed in the C57BL/6J mice, the control (or wild-type, WT) females had higher expression of ERα and stronger inhibition of ULK1 (p-ULK1^Ser757^) in vWAT than the WT males, although sWAT showed no gender difference in ERα and p-ULK1^Ser757^ (Fig. 7a, b). However, KO of ERα reduced the inhibitory phosphorylation of ULK1 (p-ULK1^Ser757^) in both sWAT and vWAT, and, most importantly, it abolished the gender difference in vWAT (Fig. 7a, b). Consistently, the gender-dependent difference in autophagy flux was diminished by the KO of ERα in vWAT (Fig. 7c–d; Figs. 4s–5s). In line with the enhanced autophagy (Fig. 7a–d; Figs. 4s–5s), which was found to promote adipogenesis (Figs. 4–5; Fig. 1s), the loss of ERα increased both sWAT and vWAT masses in the ERα KO mice compared with the WT mice (Fig. 7e–h). Furthermore, the gender difference in vWAT mass was abolished by the ablation of ERα (Fig. 7f, h). Therefore, the E2-ERα signaling cascade plays the central role in the gender difference in visceral adiposity via regulating autophagy.

## Discussion

Increased visceral adiposity has been strongly associated with higher risks of developing metabolic disorders^9–13^. Females have lower visceral fat mass than males, but this gender difference is diminished in older age groups because post-menopausal women have increased visceral adiposity; the age-related accumulation of visceral fat in post-menopausal women is likely due to drastically reduced estrogen levels^14–17,50,51^. Indeed, estrogen replacement therapy prevents post-menopausal women from excessive visceral adiposity, underlining estrogen signaling as an important regulator of visceral distribution^16,17^. To understand the underlying mechanism, we investigated the interaction between estrogen signaling and autophagy, and its relation with adipogenesis and visceral adiposity in mice. We found that female mice had lower vWAT mass than males (Fig. 1), which was associated with higher expression of ERs but lower activity of cell remodeling via autophagy in females (Figs. 2–3). The lower vWAT mass in females seemed to arise from E2-signaling suppressed autophagy and adipogenesis (Figs. 4–5, Fig. 1s). Adipogenesis was associated with downregulation of ERs and increased autophagy activity (Fig. 4, Fig. 1s). However, induction of E2 signaling dampened autophagy and adipogenesis, and use of established autophagy inhibitor BL recapitulated the effects of E2 on adipogenesis (Fig. 5, Fig. 1s). These findings suggest that the lower visceral adiposity in females is due to a stronger E2 signaling that inhibits autophagy and adipogenesis to a greater extent than in males. Indeed, ablation of ERα normalized autophagy activity and diminished the gender difference in visceral adiposity (Fig. 7, Fig. 3s). Of note, the sWAT mass in female mice was indiscernible from that in the males, although in humans females have higher sWAT adiposity than males^52–54^. The species similarity and disparity between mice and humans highlights the importance of carefully considering the strengths and limitations of rodents as physiological models for humans^55^.

The role of estrogen signaling in metabolism and adiposity has been extensively investigated^56–62^. However, this is the first study of E2 signaling in the regulation of adipocyte autophagy. Our data suggests that E2 signaling served as a suppressor of adipocyte autophagy (Figs. 4–7). In particular, E2 induced the activation of mTOR, which phosphorylated (p-ULK1^Ser757^) and thus deactivated ULK1, the key components of autophagy initiation complex (Fig. 6)^37–39^. In line with males showing lower levels of ERs in vWAT, the inhibitory phosphorylation of ULK1 by mTOR was significantly lower (2.1-fold, *p* < 0.05) than that in females (Fig. 6). Moreover, ablation of ERα significantly mitigated (*p*-ULK1^Ser757^) and normalized the gender difference in p-ULK1^Ser757^. Both *in vitro* and *in vivo* evidence supports the existence of the E2/ER-mTOR-ULK1 signaling cascade. However, further studies are warranted to determine how E2 signaling activates mTOR. In breast cancer cells it was shown that E2 might activate mTOR via small GTPase Ras homolog enriched in brain (Rheb), and it is still unclear how E2/ER signaling interacts with Rheb^63^.

Our findings may shed light on the increased visceral adiposity and metabolic syndrome (e.g., fatty liver) in breast cancer patients receiving anti-estrogenic treatment^64,65^. For instance, tamoxifen, a selective ER modulator that binds to ERs and suppresses estrogen action, was found to significantly increase body mass index (30.9 on average, indicative of obesity), visceral fat area, and incidence of type 2 diabetes in women with breast cancer^64^. The E2–autophagy–adipogenesis axis identified in this study may account, at least in part, for the tamoxifen-induced visceral obesity in tamoxifen users. In laboratory animals, hyperplasia (i.e., increased adipogenesis) can be induced by removal of E2 (via ovariectomy) or ERα (via genetic KO) which increases visceral fat mass and impairs metabolism; by contrast, administration of E2 reduces adiposity and improves metabolic homeostasis^61,62,66,67^. Therefore, the effects of E2–autophagy axis on adiposity and metabolic homeostasis should be taken into consideration in future anti-estrogenic treatment of breast cancer.

Taken together, our study provides the first line of evidence that E2/ER signaling mediates gender difference in visceral adiposity by dampening autophagy and adipogenesis via the mTOR-ULK1 pathway. The males have distinctly lower expression of ER in visceral fat than the females, thereby enhancing autophagy and adipogenesis and leading to higher distribution of visceral fat in male mice. Although ERβ cannot be excluded from the regulating process, ERα appears to play the dominant role because deletion of ERα alone normalized the gender differences in autophagy activity and visceral adiposity. This study adds to the importance of considering the gender perspective on the role of autophagy in human diseases^68^. Given that nutrient signal also regulates mTOR activity^38^, it would be of interest for future investigation to depict how E2/ER interacts with nutrient statuses (e.g., fasting or feeding with high-energy diet) in the regulation of autophagy and adiposity.

## Materials and methods

### Mice

C57BL/6J mice were housed in plastic cages on a 12-h light–dark photocycle and with free access to water and regular chow diet as described previously^25,34^; at the age of 6–7 week old, the mice were weighed and sacrificed for tissue collection. The WT and global ERα KO mice were obtained by breeding heterozygous males to females as described previously^49^; at the age of 12–16 week old, the WT and ERα KO mice were weighed and sacrificed for tissue collection. The WAT pads were collected and weighed quickly before SVF isolation, explant culture for autophagy flux analysis, or snap freezing in liquid nitrogen. Animal use procedures followed the National Institutes of Health guidelines and were approved by the Virginia Tech Institutional Animal Care and Use Committee.

### 3T3L1 cell culture, differentiation, and treatment

3T3L1 preadipocytes (ATCC CL-173, Manassas, VA, USA) were cultured in basal media (DMEM media containing 10% FBS, 100 units/ml penicillin, and 100 μg/ml streptomycin (1× P/S)), at 37 °C in a humidified atmosphere of 5% CO_2_^33–35^. The media were replaced every 2 days until the cells became confluent (day 0), and after 2 more days (day 2) the medium was changed to differentiation medium I (DMEM with 10% FBS, P/S (1×), IBMX (0.5 mM), dexamethasone (1 μM), insulin (1 μg/ml), and rosiglitazone (2 μM)). At the end of day 4, the medium was changed to differentiation medium II (DMEM with 10% FBS, P/S (1×), and insulin (1 μg/ml)). At the end of day 6, the medium was changed to basal media and the cells were maintained until day 12. Preadipocytes without differentiation induction were maintained in basal media and supplied with fresh medium every 2 days till day 12. E2 at the concentrations of 1 nM–10 μM has been used to treat adipocytes^69–76^. Our preliminary tests indicated that E2 at 1 nM, 10 nM, 0.1 μM, and 0.2 μM imposed similar effects, but E2 at 0.1 μM (likewise 0.2 μM) was the most potent (data not shown). As such, we used E2 of 0.1 μM for the treatments starting on day 0 through day 12. Other chemicals were used at the concentrations established previously, including PPT (0.1 μM), DPN (0.1 μM), and bafilomycin A1 (4 nM), and leupeptin (0.4 ng/ml)^25,77,78^, to treat the cells during differentiation.

### Primary stromal vascular cell culture, differentiation, and treatment

Fresh gonadal WAT from C57BL/6J mice were dissected, minced, and digested as previously described^79^. Cells were suspended in basal media (DMEM/F12 containing 10% FBS and 100 units/ml penicillin and 100 μg/ml streptomycin (1× P/S)), and centrifuged at 500 × *g* for 5 min. The pellet was resuspended in basal media and filtered through a 40-micron cell strainer. After centrifugation (500 × *g*) for 5 min, the pellet was resuspended in basal media and plated on 10-cm dishes. After cell subculture to a 95% confluence (day 0) on 6-well plates, differentiation was induced in differentiation medium (DMEM/F12 medium with 10% FBS, 1× Pen/Strep, dexamethasone (5 μM), insulin (0.5 μg/ml), IBMX (0.5 mM), and rosiglitazone (1 μM)) for 4 days (day 4). Then the cells were maintained in maintenance medium (DMEM/F12 medium containing 10% FBS, 1× Pen/Strep, and insulin (0.5 μg/ml)) for 6 days (day 10). The treatments with chemicals (E2 at 0.1 μM, bafilomycin A1 at 4 nM, and leupeptin at 0.4 ng/ml) started on day 0 through day 10 to examine their effects on autophagy and adipogenesis.

### Oil Red O staining

The Oil Red O working solution was freshly prepared by mixing 0.35% stock solution with dH_2_O (6:4) and filtered, and the staining was conducted as described^25,33,35^. After the media were removed, the cells were washed once with cold phosphate buffered saline, and fixed in 4% formaldehyde at room temperature for 10 min. The cells were then washed with dH_2_O and air dried. Oil Red O working solution was added to start the staining at room temperature for 1 h. The stained cells were washed with dH_2_O for four times before the images were captured with a Nikon ECLIPSE Ti Inverted Microscope (Melville, NY, USA).

### Autophagy flux assay

To measure autophagy flux in cultured cells, we treated 3T3L1 preadipocytes, stromal vascular cells, and mature adipocytes (day 10) with bafilomycin A1 (inhibitor of autophagosome acidification, at 0.1 μM) plus leupeptin (the inhibitor of lysosomal proteases, at 10 μg/ml) for 4 h. The cells were then harvested to prepare cell lysates as previously described^25,33,35^. To measure autophagy flux in WAT explants, freshly collected adipose tissues were minced into small tissue fragments (2–3 mm^3^) and cultured for 4 h with DMEM medium supplemented with 2 mM glutamine, 1% (vol/vol) antibiotic solution, and 10% (vol/vol) FBS in a CO_2_ incubator (37 °C, 5% CO_2_). The WAT explant cultures in the presence or absence of bafilomycin-A1 (0.1 μM) and leupeptin (10 μg/ml) were then harvested and lysed as described previously^25^. The turnover of LC3-II or p62 protein, i.e., the substrates of autophagy for degradation, was measured by Western blotting and image analysis to assess autophagy flux^25,27,28^.

### Western blotting

Tissue and cell lysates were prepared with PLC lysis buffer (30 mM Hepes, pH 7.5, 150 mM NaCl, 10% glycerol, 1% Triton X-100, 1.5 mM MgCl_2_, 1 mM EGTA, 10 mM NaPPi, 100 mM NaF, 1 mM Na_3_VO_4_) supplemented with protease inhibitor cocktail (Roche), and 1 mM PMSF^80^. Total protein concentrations of the lysates were determined using a DC protein assay kits (Bio-Rad). Antibody (catalog number) information: GAPDH (MA5-15738) and β-actin (MA5-15739) antibodies were purchased from Pierce (Rockford, IL, USA); Atg5 (12994s), Atg7 (8558s), Atg12 (2011s), p62 (5114s), p-mTOR (Ser2448) antibody (5536s), and p-ULK1 (Ser757) antibody (14202s) from Cell Signaling Technology (Beverly, MA, USA); Beclin 1 (MABN16), ERα (04–820), and ERβ (GR39) antibodies from EMD Millipore (Billerica, MA, USA); and LC3B antibody (L7543) from Sigma.

### Statistical analysis

Data are presented as mean ± SD. Differences between the groups were validated by one-way ANOVA with the least significant difference post hoc test to detect statistical differences between groups and treatments (DI+ vs. DI−, E2+ vs. E2−, and BL+ vs. BL−). Differences in autophagy and adipose parameters between males and females were validated by a *t*-test. A value of *p* < 0.05 was considered statistically significant.

## Electronic supplementary material

supplemental data
