# Supplementary material for: Estradiol signaling mediates gender difference in visceral adiposity via autophagy
Source: Cell Death Dis. 2018 Feb 22;9(3):309. doi: 10.1038/s41419-018-0372-9 (PMC5833393; doi:10.1038/s41419-018-0372-9)

**Figure 1s**. Estradiol dampened autophagy and differentiation of primary stromal vascular cells. (A-B) The presence of E2 (0.1 μM, days 0-10) suppressed autophagy activity during differentiation of primary stromal vascular cells. The autophagy flux was analyzed by Western blotting (A) and densitometry (B) after the cells were incubated with and without autophagy inhibitor BL (bafilomycin A1 at 0.1 μM and leupeptin at 10 μg/ml) for 4 hours on day 10. DI+ represents stromal vascular cells with differentiation induction and harvested on day 10; DI- represents stromal vascular cells without differentiation induction and harvested on day 10. For autophagy flux analysis, we first normalized the band densities of p62 against that of β-actin, then calculated the differences of normalized densities in the presence vs. the absence of autophagy inhibitor; lastly, the differences were shown as fold changes by taking the DI-E2- group as “1”. (C) Estradiol (0.1 μM, days 0-10) suppressed the differentiation of primary stromal vascular cells, which was phenocopied by the treatment with autophagy inhibitor bafilomycin A1 (4 nM) and leupeptin (0.4 ng) during day 0 through day 10. Oil red O staining was conducted on day 10. *, p<0.05; **, p<0.01; n=3-4.

**Figure 2s**. Effects of the estradiol-ER signaling on autophagy proteins. (A-B) E2 (0.1 μM, days 0-12) had marginal effects on beclin 1, Atg5, Atg7, and Atg12-Atg5 conjugate, analyzed by Western blotting (A) and densitometry (B). DI+ represents 3T3L1 cells with differentiation induction and harvested on day 12; DI- represents 3T3L1 cells without differentiation induction and harvested on day 12. (C-D) The females and males showed similar patterns of beclin 1, Atg5, Atg7, and Atg12-Atg5 conjugate, analyzed by Western blotting (C) and densitometry (D). In densitometric analysis, the band densities of investigated proteins were normalized against that of GAPDH, and the fold changes were calculated by taking the normalized density of DI-E2- group (panels A and B) or the female group (panels C and D) as “1”. **, p<0.01; n.s., not significant; n=3-4.

**Figure 3s.** Effects of PPT and DPN on autophagy and adipogenesis. PPT (0.1 μM) or DPN (0.1 μM) was used at the established concentrations[^76^](#_ENREF_76)^,^ [^77^](#_ENREF_77) during 3T3L1 preadipocyte differentiation (days 0-12). DI+ represents 3T3L1 cells with differentiation induction and harvested on day 12. Autophagy flux (i.e., the turnover of LC3-II or p62) was analyzed by Western blotting (A) and densitometry (B) after the cells were incubated with and without autophagy inhibitor BL (bafilomycin A1 at 0.1 μM and leupeptin at 10 μg/ml) for 4 hours. For autophagy flux analysis, we first normalized the band densities of LC3-II and p62 against that of GAPDH, then calculated the differences of normalized densities in the presence vs. the absence of autophagy inhibitor; lastly, the differences in autophagy flux were shown as fold changes by taking the DI+ group as “1”. n= 4; *, p<0.05; **, p<0.01. (C) The presence of PPT but not DPN (days 0-12) suppressed the differentiation of 3T3L1 preadipocytes, validated by oil red O staining on day 12.

**Figure 4s**. Measurements of autophagy flux by LC3-II turnover in wild type (WT) and ERα knockout (KO) mice. (A) Representative images of Western blotting assay for autophagy flux in sWAT from WT mice. (B) Representative images of Western blotting assay for autophagy flux in vWAT from WT mice. (C) Representative images of Western blotting assay for autophagy flux in sWAT from KO mice. (B) Representative images of Western blotting assay for autophagy flux in vWAT from KO mice. The results of densitometric analysis were shown in Figure 7. BL, bafilomycin A1 and leupeptin; M, male; F, female.

**Figure 5s**. Measurements of autophagy flux by p62 turnover in wild type (WT) and ERα knockout (KO) mice. (A) Representative images of Western blotting assay for autophagy flux in sWAT from WT mice. (B) Representative images of Western blotting assay for autophagy flux in vWAT from WT mice. (C) Representative images of Western blotting assay for autophagy flux in sWAT from KO mice. (B) Representative images of Western blotting assay for autophagy flux in vWAT from KO mice. The results of densitometric analysis were shown in Figure 7. BL, bafilomycin A1 and leupeptin; M, male; F, female.


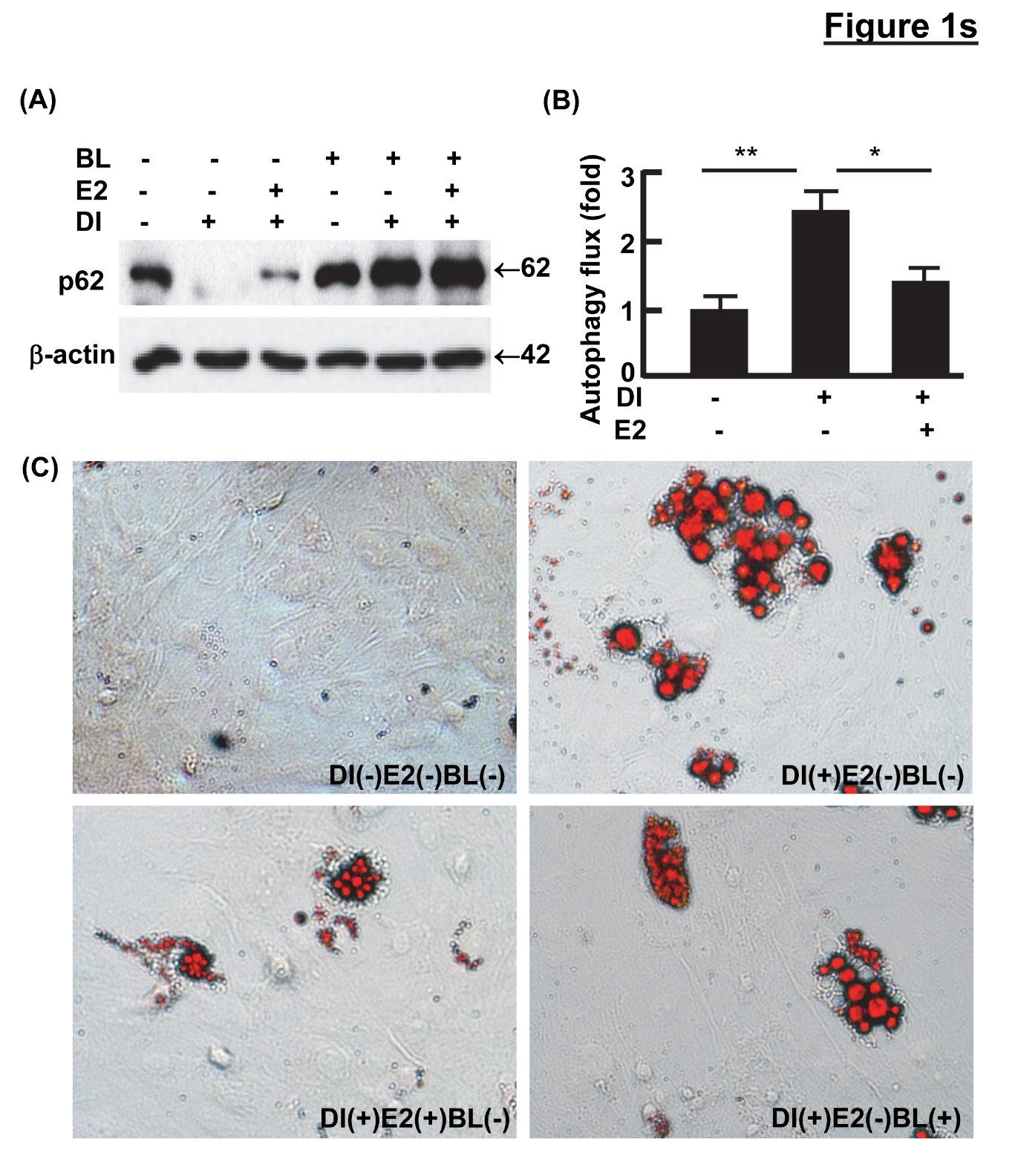


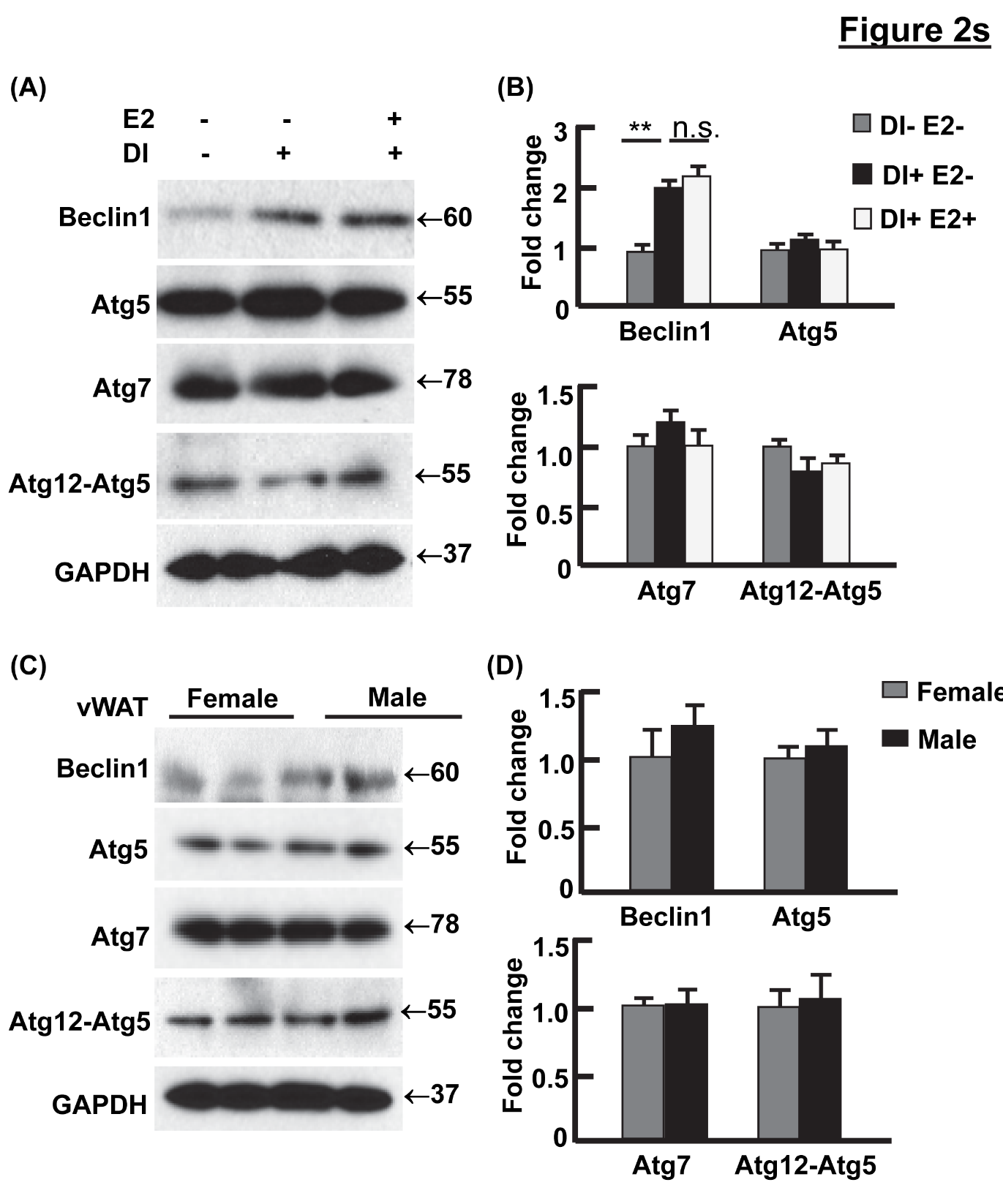


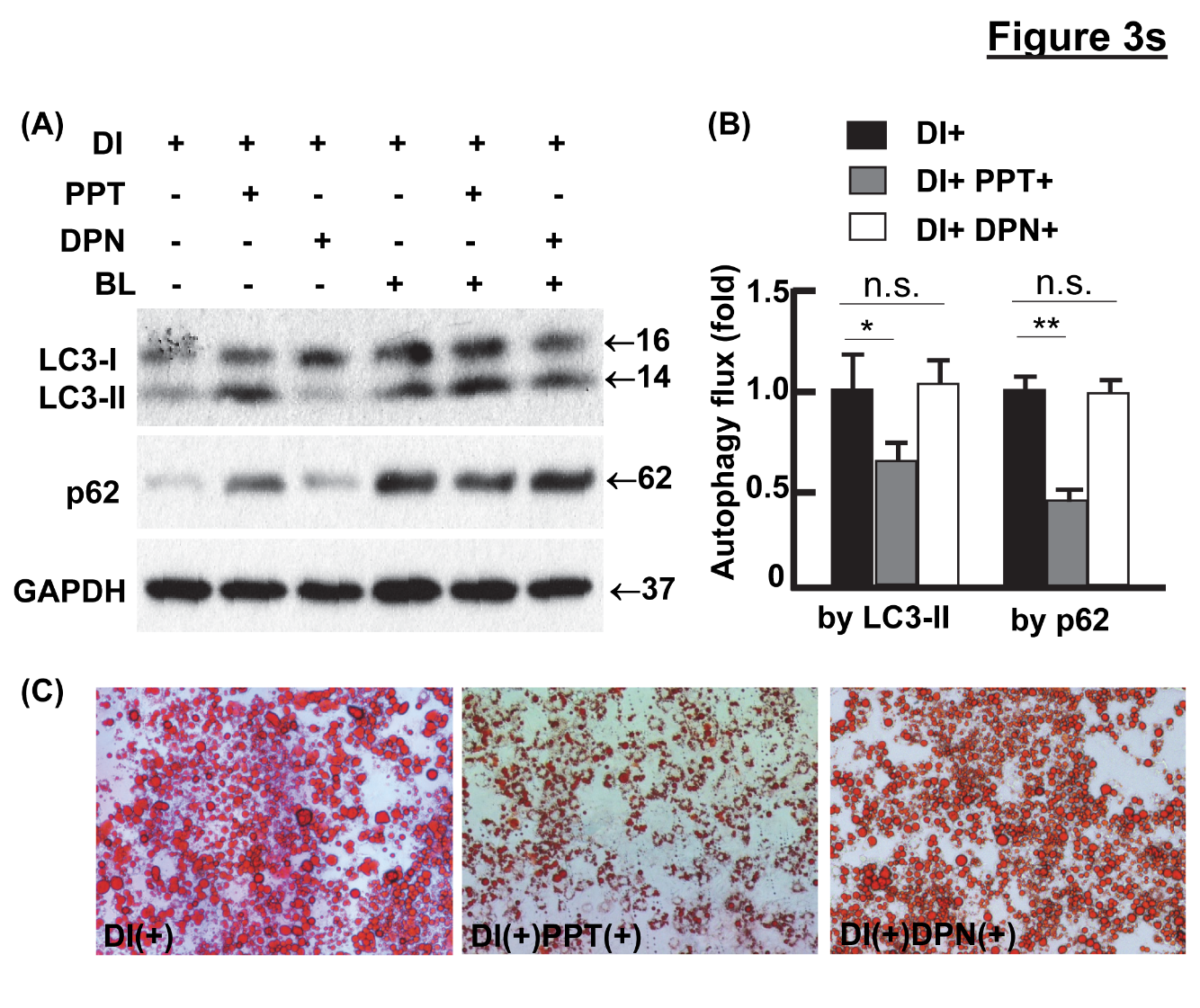


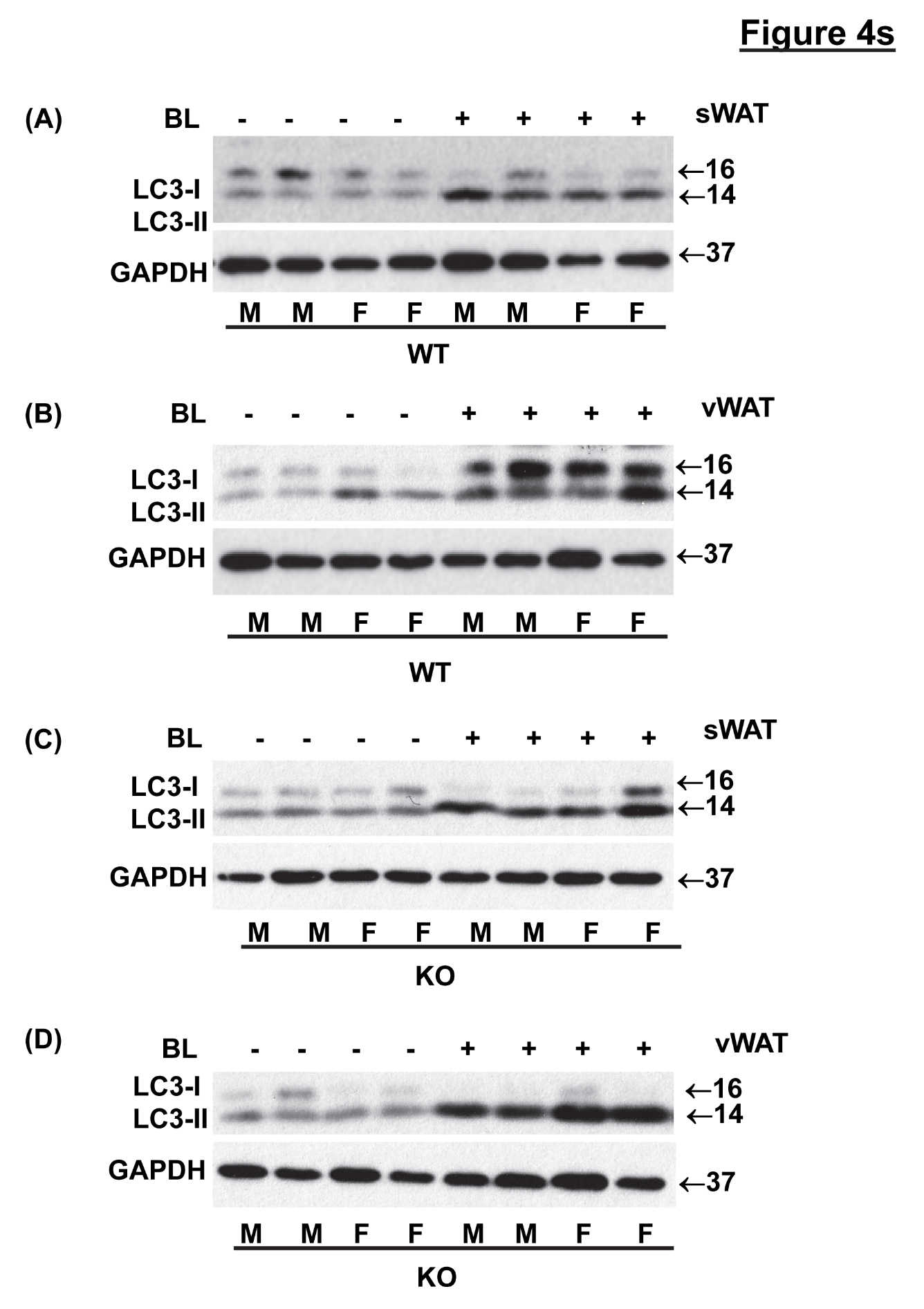


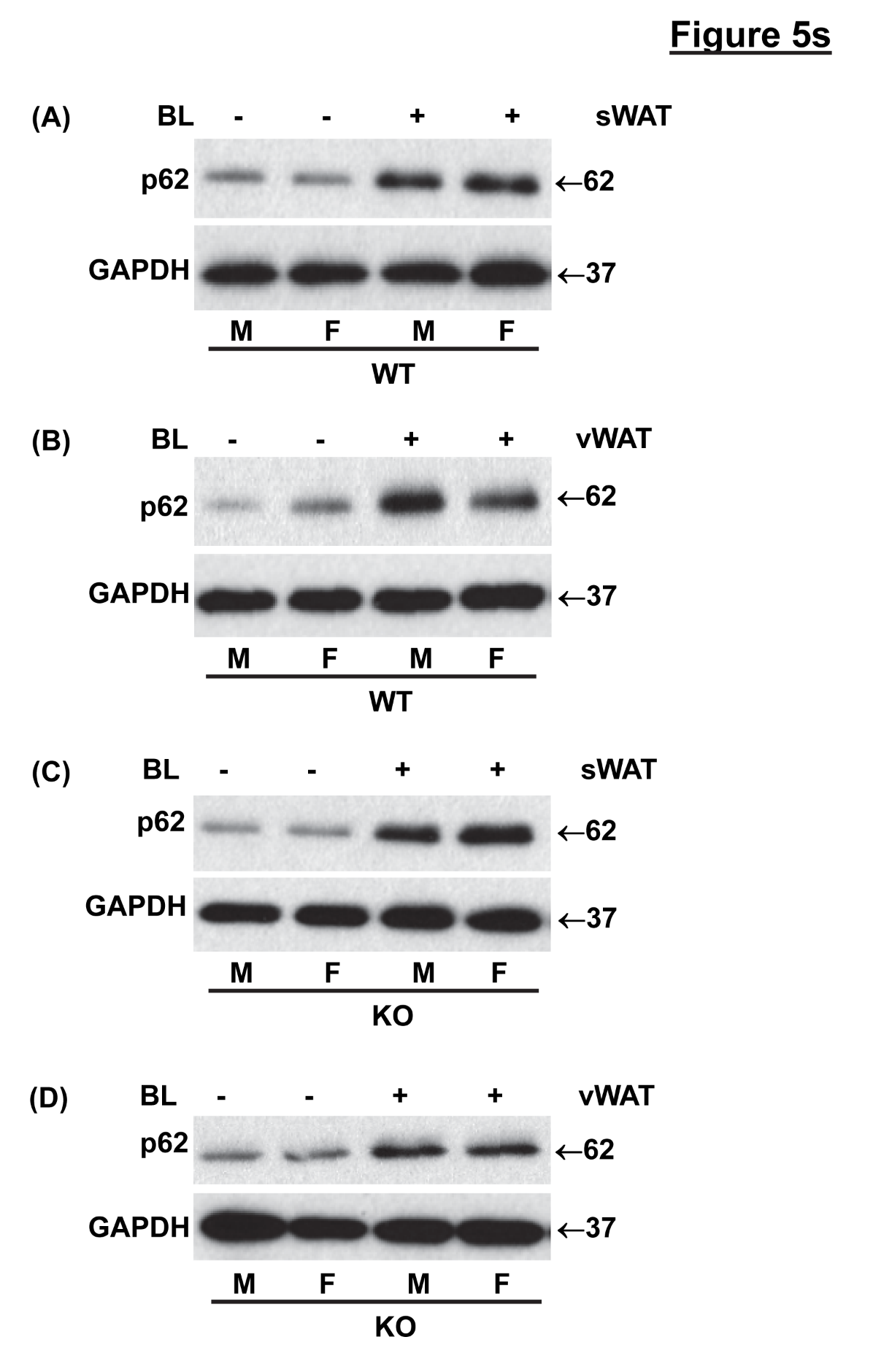

Supplement: Supplementary file 1 — supplemental data [file 41419_2018_372_MOESM1_ESM.docx]
